# Artery of Percheron Infarction: A Case Report of Bilateral Thalamic Stroke Presenting with Acute Encephalopathy

**DOI:** 10.1155/2022/8385841

**Published:** 2022-03-30

**Authors:** Charles Donohoe, Nooshin Kiani Nia, Patricia Carey, Vamsi Vemulapalli

**Affiliations:** ^1^Department of Neurology Chair, Truman Medical Center, 2301 Holmes St, Kansas City, MO 64108, USA; ^2^Department of Neurology, Truman Medical Center, 2301 Holmes St, Kansas City, MO 64108, USA; ^3^University of Missouri-Kansas City School of Medicine, 2411 Holmes St, Kansas City, MO 64108, USA

## Abstract

The artery of Percheron (AOP) is a relatively rare anatomic variant in which a solitary arterial trunk branches from the proximal segment of the posterior cerebral artery and provides arterial supply to the paramedian region of the thalami bilaterally and often to the rostral part of the midbrain. Occlusion of the artery of Percheron results in bilateral paramedian thalamic infarcts with and without midbrain involvement. Recognition of this condition as an acute stroke may be challenging due to various nonlocalized clinical presentations, given the wide range of neurological functions subserved by the thalamus. Prompt neuroimaging, preferably with magnetic resonance imaging (MRI), in conjunction with familiarity with this relatively rare vascular variation can facilitate initiation of appropriate time contingent thrombolytic treatment and improved long-term prognosis. We present a case of a 56-year-old African American female with a bilateral thalamic infarct secondary to the artery of Percheron thromboembolism. This patient presented unresponsive without focal neurologic findings but with an initial Glasgow Coma Score (GCS) of 7, and subsequent computed tomographic (CT) head revealed bilateral thalamic hypodensities. Confirmatory MRI exhibited bilateral subacute thalamic infarcts, which were thought to be embolic with the source from the left ventricular thrombus as the patient had at least three distinct clots. Unfortunately, the patient's mental status did not improve significantly, and she was discharged to a nursing facility for extended care. AOP infarction may be missed on vascular imaging utilizing CT, MRI, and even catheter angiography. Clinical recognition that the AOP is one of the only single artery occlusions that can affect bilateral structures and frequently present solely as altered mental status without focal neurologic deficits is crucial to the diagnosis.

## 1. Background

According to Gerald Percheron, who studied thalamic blood supply extensively in the twentieth century, the P1 segment of the posterior cerebral artery (PCA) supplies the paramedian thalamus through the paramedian arteries on each side [1, 2]. Paramedian arteries may sometimes arise from one common trunk from a P1 segment on either side: this common trunk is known as the artery of Percheron [2]. A bilateral paramedian thalamic infarct with or without the involvement of the rostral midbrain can result if the blood supply to the AOP is compromised [2]. Classic findings of this condition include impaired arousal, memory, or language impairment, and occasionally an ocular movement disorder [3, 4]. AOP infarctions are rarely associated with pyramidal symptoms, and initial noncontrast CT head imaging can be normal and misleading [5].

**Figure 1:**
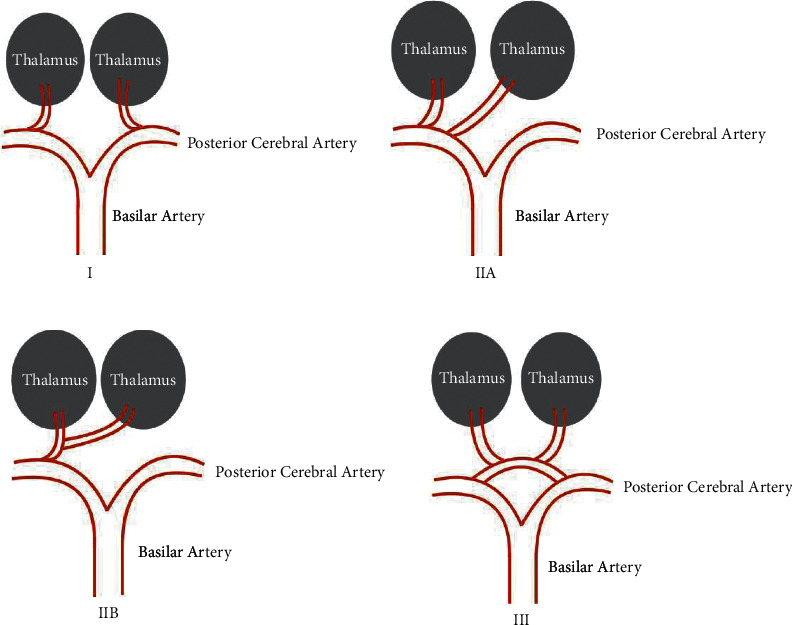
Anatomy of thalamic arterial supply variants.

These varied clinical presentations without localizing findings and the rarity of this anatomic variant often cause significant delay in obtaining MR brain imaging and ultimately identifying an accurate diagnosis [5].  Figure 1 demonstrates four variations in the arterial supply of the thalamic circulation, and Table 1 provides a summarized description of each thalamic arterial supply variant. Variant I (tuberothalamic territory): this is the most common variant, where the perforating arteries on each side will arise from each right and left posterior communicating artery (PCA). Vascular compromise leads to impaired orientation, arousal, memory, learning, personality, and executive dysfunction. Variant IIA (inferolateral territory): the proximal segment of one PCA gives rise to perforating arteries in an asymmetric pattern. The perforating arteries arise from the proximal segment of one PCA. Vascular compromise will lead to hemisensory loss, hemiparesis, hemi-ataxia of the contralateral side, and poststroke hyperalgesia, particularly with right-sided strokes. Variant IIB (posterior choroidal territory): bilateral perforating thalamic arteries arise from a single arterial trunk referred to as the artery of Percheron. Vascular compromise leads to sensory loss of the contralateral side, visual field defects, tremor, dystonia, weakness, and occasional language impairment. Variant III (paramedian territory): several small perforating branches arise from one arterial supply that bridges the P1 segments of both posterior communication arteries. Vascular compromise leads to hypersomnolence if the lesion is bilateral in addition to symptoms such as memory impairment, difficulty in learning, and language deficits [5].

## 2. Case Presentation

A 56-year-old African American female with a past medical history of nonischemic cardiomyopathy, hypertension, chronic obstructive pulmonary disease (COPD), hyperlipidemia, left ventricular thrombus on rivaroxaban, depression, anxiety, and polysubstance use disorder was found unresponsive at a house frequented by known drug abusers. She presented to the emergency room with acute encephalopathy of unknown cause. On admission, the patient had a GCS of 7, which improved with the administration of 2 milligrams of naloxone for concern of drug use as the cause of her encephalopathy. The patient did not have any focal neurologic findings, and no stroke alert protocol was initiated.

The patient then intermittently desaturated to 84% oxygen saturation levels with an episode of apnea and was subsequently placed on 3–4 liters of oxygen through a nasal cannula. As she was able to protect her airway, she was not intubated. In addition, the patient was hypertensive with a blood pressure of 152/100 mmHg on arrival.

The initial basic metabolic panel in the emergency department was remarkable for hyponatremia at 133 mEq/L, but otherwise, the electrolytes were within normal limits. Further blood testing demonstrated an elevated creatinine kinase level of 1281 U/L and alcohol, salicylate, and acetaminophen levels within reference ranges. A urine drug screen was positive for amphetamines, cocaine, and cannabinoids.

**Figure 2:**
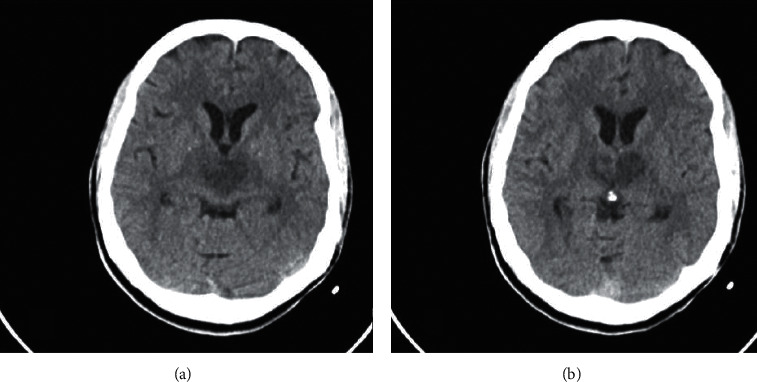
Noncontrast CT brain demonstrating bilateral hypodensities in the medial thalami obtained at the time of admission.

**Figure 3:**
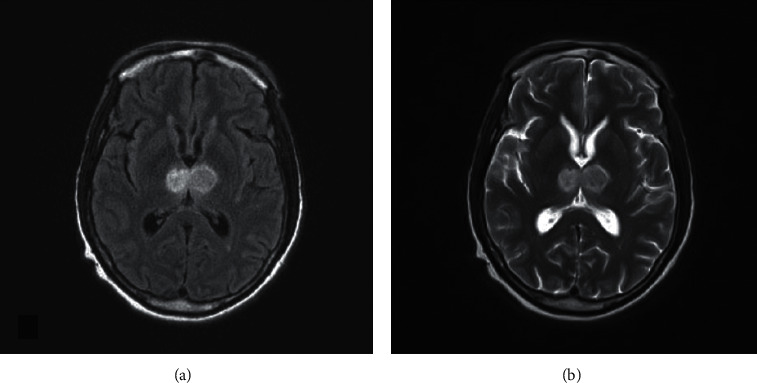
Axial flair (a) and axial T2 (b) MR images obtained one week after the CT demonstrated increased signal bilaterally in the thalamic nuclei.

**Figure 4:**
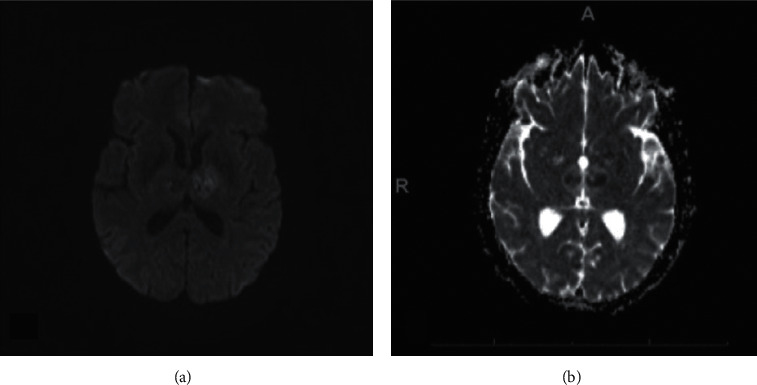
Axial DWI (a) and axial ADC (b) demonstrate subacute ischemic infarct bilaterally in the thalamus with restricted diffusion and low ADC values.

**Figure 5:**
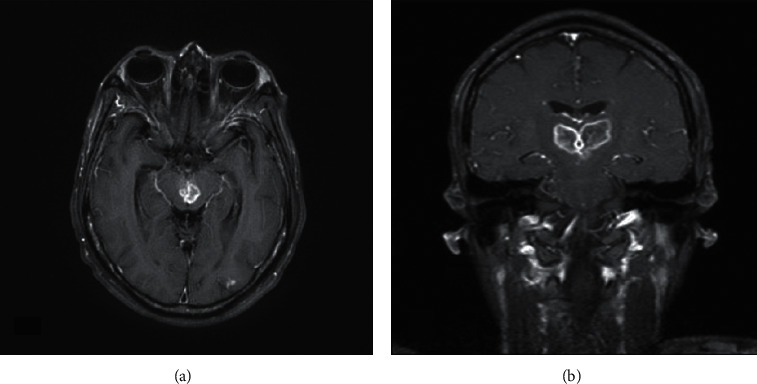
Postcontrast T1 MR images demonstrate vivid contrast enhancement involving the midbrain: (a) axial image and posterior thalami; (b) coronal image.

CT head without contrast revealed hypodensity in both thalami, which prompted a teleneurology consult and they recommended that tissue plasminogen activator (TPA) not be administered because patient was taking rivaroxaban and the time last known well was unclear. CT angiogram (CTA) did not identify an isolated vessel occlusion. MRI brain confirmed bilateral thalamic infarcts. MR following gadolinium administration demonstrated vivid enhancement in both thalami and upper midbrain—characteristic of a subacute infarction (Figures 2–5). MR venogram (MRV) demonstrated a patent intracranial venous system (not shown).

An echocardiogram was then performed, which showed an ejection fraction of 20–25% and three distinct clots in the anterior, lateral, septal, and apical walls that were all more than 1 cm in size. On that basis, the thalamic infarcts were believed to be embolic, with the source as a left ventricular thrombus.

The patient's mental status failed to improve over her 48 days of hospital stay, and her Mini-Cog evaluation measured 0/5. She ultimately developed dysphagia requiring Percutaneous Endoscopic Gastrostomy (PEG) tube placement. Guardianship was instituted due to the patient's incapacity, and she was discharged to a skilled nursing facility for extended care.

## 3. Discussion

The AOP is the IIB variant of the thalamic circulation, where a single arterial trunk gives rise to bilateral perforating thalamic arteries [6]. Therefore, any compromise in the AOP will lead to compromised circulation to bilateral thalami with or without midbrain involvement [6]. The AOP can rarely be demonstrated on MRA, CTA, or conventional catheter angiography due to its small diameter, rendering it challenging to visualize. Therefore, confirmation by vascular imaging of the precise vessel occlusion is not a diagnostic requirement [7]. The AOP variant prevalence is estimated to occur in 4–30% of the general population and accounts for between 0.1% and 2% of ischemic strokes [1, 2, 8–11]. The bilateral and frequently symmetrical imaging abnormalities in the thalami and midbrain in conjunction with prominent mental status findings often initially point to a metabolic or toxic etiology, delaying the diagnosis of an uncommon stroke syndrome [3, 11].

The AOP infarction is an imaging diagnosis. MRI is the preferred modality approaching 100% sensitivity, while, unlike our patient, CT is frequently negative (50% sensitivity). Early diagnosis is best made with diffusion-weighted imaging (DWI) with apparent diffusion coefficient (ADC) sequences [11]. The AOP occlusion on CTA or MRA is an unlikely finding and not a diagnostic prerequisite [7, 12]. Thrombolysis is the treatment of choice when a diagnosis is made within the therapeutic window of 4.5 hours. Because of the nonlocalizing presentations associated with the change in mental status, many diagnoses are delayed and fall outside the therapeutic window. Prognosis can be favorable if the diagnosis is rapidly identified, and the patient receives appropriate treatment [7].

A series of 18 cases of paramedian infarction reported that 61% of them had favorable outcome and were able to perform activities of daily living without assistance [13]. There have been reported cases of bilateral thalamic strokes, who presented mainly with sudden onset of hypersomnia and fluctuating arousal as the thalamus plays an important role in sleep regulation and in maintaining arousal. Hypersomnolence can be due to the interruption of noradrenergic and dopaminergic pathways from the ascending reticular activating system to the thalamus [14–16].

One case report stated that there was significant improvement in the state of alertness after administering modafinil 100 mg twice a day [8]. In our case, we report a patient who was found unresponsive with a GCS of 7. Initial CT head without contrast showed abnormal hypodensity throughout the bilateral thalami consistent with an AOP infarction. MRI brain with and without contrast was performed and confirmed the earlier findings. MRV ruled out dural venous sinus thrombosis.

Unfortunately, this patient was outside the therapeutic window for thrombolysis. Her echocardiogram showed at least three distinct clots in the anterior, lateral, septal, and apical walls, all more than 1 cm in size. The thalamic infarcts were thought to be embolic from the source of a left ventricular thrombus, and she was started on warfarin. The role of multiple illicit substances including cocaine was considered a potential contributing factor. Due to the severity of the stroke and the corresponding clinical presentation, the prognosis of this patient was unfortunately poor, and she was supported throughout her hospital stay without significant clinical improvement.

## 4. Conclusion

Occlusion of the AOP can result in various clinical presentations, including acute encephalopathy, cognitive impairment, oculomotor defects, motor dysfunction, sensory symptoms, and behavioral abnormalities. In the setting of bilateral thalamic imaging abnormalities, a broad differential must be investigated, including cerebral venous thrombosis, basilar artery thrombosis, carbon monoxide toxicity, hypoxic ischemic encephalopathy, osmotic demyelination, Wernicke's encephalopathy, rabies, and flavivirus (Japanese) encephalitis. Demonstration of AOP occlusion by vascular imaging studies including MR angiography, CT angiography, and conventional angiography can be insensitive due to the small diameter of the AOP. Therefore, negative vascular imaging studies (MRA, CTA, and conventional catheter angiography) do not rule out the diagnosis. Timely diagnosis and treatment are facilitated by awareness of the diverse nonstroke-like clinical presentations and characteristic neuroimaging findings—improving the management of the infrequently encountered patient with an AOP infarction.

## Figures and Tables

**Table 1:** Description of thalamic arterial supply variants.

Type	Anatomical description
I	Normal paramedian artery anatomy: L and R PMAs arise from their respective PCA
IIA	Variant of paramedian artery anatomy: both PMAs arise from either L or R PCA
IIB	Artery of Percheron anatomy: one branch from either L or R PCA supplies both thalami
III	Variant of paramedian artery anatomy: both PMAs arise from an arterial branch connecting the L and R PCA

## Data Availability

The data used to support the findings of this study are included within the article under references and cited throughout the body of the article with a corresponding reference in the reference list.

## Abbreviations

AOP: Artery of Percheron
GCS: Glasgow Coma Score
CT: Computed tomography
CTA: Computed tomography angiography
MR: Magnetic resonance
MRI: Magnetic resonance imaging
MRA: Magnetic resonance angiography
CTA: Computed tomography angiography
PCA: Posterior cerebral artery
COPD: Chronic obstructive pulmonary disease
PEG: Percutaneous endoscopic gastrostomy
DWI: Diffusion weighted imaging
ADC: Apparent diffusion coefficient.
